# Long-Day Photoperiod Improves the Growth and Muscle Quality of Grass Carp (*Ctenopharyngodon idella*)

**DOI:** 10.3390/foods14030504

**Published:** 2025-02-05

**Authors:** Yin Wang, Xuxu Li, Tingting Xu, Huacheng Li, Jieya Liu, Qiushi Yang, Wenhan Li, Sayed R. S. Zidan, Chengchen Jiang, Yutian Yuan, Rong Tang, Liqin Yu, Li Li, Xi Zhang, Dapeng Li

**Affiliations:** 1 Hongshan Laboratory, College of Fisheries, Huazhong Agricultural University, Wuhan 430070, China; wyin@webmail.hzau.edu.cn (Y.W.); lixuxu@webmail.hzau.edu.cn (X.L.); 2021308110042@webmail.hzau.edu.cn (T.X.); lihuacheng@webmail.hzau.edu.cn (H.L.); 120160001@taru.edu.can (J.L.); y357446@webmail.hzau.edu.cn (Q.Y.); lwh@webmail.hzau.edu.cn (W.L.); srs11@fayoum.edu.eg (S.R.S.Z.); chengchenjiang@webmail.hzau.edu.cn (C.J.); yuanyutian@webmail.hzau.edu.cn (Y.Y.); tangrong@mail.hzau.edu.cn (R.T.); yuliqin@mail.hzau.edu.cn (L.Y.); foreverlili78@mail.hzau.edu.cn (L.L.); zhangxi@mail.hzau.edu.cn (X.Z.); 2 Engineering Research Center of Green Development for Conventional Aquatic Biological Industry in the Yangtze River Economic Belt, Ministry of Education, Wuhan 430070, China; 3 Hubei Provincial Engineering Laboratory for Pond Aquaculture, Wuhan 430070, China; 4 College of Life Sciences and Technology, Tarim University, Alar 843300, China; 5 Animal Production Department, Faculty of Agriculture, Fayoum University, Fayoum 63514, Egypt

**Keywords:** *Ctenopharyngodon idella*, photoperiod, growth, muscle fibers, muscle quality

## Abstract

To investigate the effects of photoperiods on the growth and muscle quality indicators of grass carp (*Ctenopharyngodon idella*), 225 fish (109.65 ± 3.62 g) were randomly assigned into five different photoperiod groups (0L:24D, 8L:16D, 12L:12D, 16L:8D, and 24L:0D). The experiment spanned a 75-day period, after which sampling and analysis were performed. Compared with the 0L:24D and 8L:16D groups, the 12L:12D and 16L:8D groups significantly promoted the growth of grass carp (*p* < 0.05). The texture parameters of the muscle in the 0L:24D and 16L:8D groups were significantly greater than those in the 12L:12D group (*p* < 0.05). The crude protein content was significantly higher in the 12L:12D and 16L:8D groups (*p* < 0.05). The amino acid content and muscle fiber characteristics, as well as the mRNA levels of myostatin (*mstn*), myogenic factor 5 (*myf5*), type I collagen α1 (*col1α1*), and α2 (*col1α2*), along with the hydroxyproline and collagen contents, were all significantly influenced by the photoperiod (*p* < 0.05). The lysine (Lys), aspartic acid (Asp), and alanine (Ala) contents in the muscle and muscle fiber density of grass carp reached the highest levels under the 16L:8D treatment (*p* < 0.05). Collectively, these results indicate that a 16L:8D photoperiod is optimal for enhancing both the growth and muscle quality indicators of grass carp. The findings of this study offer valuable scientific references for the precise regulation of grass carp quality when using a photoperiod, and they are anticipated to foster the further development and optimization of strategies for improving grass carp quality.

## 1. Introduction

With the improvement of living standards, there has been an increasing demand for high-quality protein foods, which has led to growing attention to aquatic animals as food resources. The apparent consumption of aquatic animal-based food reached 162.5 million tons in 2021. During this time span, the global per capita annual consumption has risen from 9.1 kg in 1961 to 20.7 kg in 2022. Notably, 89% of aquatic animal production is utilized for direct human consumption. In 2021, out of the per capita protein supply of 3.2 billion people, aquatic animal food proteins accounted for at least 20% of the total animal-derived proteins, indicating that aquaculture play a key role in ensuring global food security [1]. In various types of animal-based foods, fish are known for their high protein content, low lipid level, and richness in essential amino acids and polyunsaturated fatty acids [2]. In this context, how to improve the muscle quality of fish while increasing yield has also become a focus in the field of food research. The quality of fish meat is defined by sensory characteristics, chemical composition, and physical properties, which influence consumers’ acceptance of fish [3]. Throughout the farming process, the primary external determinants of fish meat quality include food and the ecological environment [4,5]. Among them, the photoperiod serves as a crucial ecological determinant for cultured fish, influencing the entire life cycle of teleost fish. The successful application of photoperiod manipulation has been observed in enhancing growth and muscle quality across a multitude of fish species. For instance, continuous illumination has been observed to soften the texture of Atlantic cod (*Gadus morhua*) muscles [5] while not affecting its protein and lipid content [6]. Conversely, it also significantly influences the growth of Atlantic salmon (*Salmo salar*), leading to an increase in muscle fiber density and firmness [7]. In juvenile European seabass (*Dicentrarchus labrax*), 8L:16D promotes peak values in the daily weight gain coefficient and total amino acid content [8]. These studies revealed that the differential impacts of the photoperiod on growth indices and meat quality is species-dependent, and it may be influenced by the duration of photoperiod exposure.

Grass carp (*Ctenopharyngodon idellus*) is an important freshwater edible fish in the world, and it is favored by consumers due to its tenderness, flavor, comprehensive nutrition, and reasonable price, as well as due to providing low-cost animal protein for people worldwide, especially in developing countries and underdeveloped areas [9]. Despite extensive research on the influence of various environmental factors on the muscle quality of grass carp [2,10,11], there remains a lack of studies examining the impact of photoperiod on their growth performance and nutritional quality. To address the existing research gap and enhance the muscle quality of grass carp by refining breeding conditions, our experiment selected five photoperiod treatments (0L:24D, 8L:16D, 12L:12D, 16L:8D, and 24L:0D) to assess their effects on the growth and muscle quality indicators of grass carp. This study aims to provide a reliable reference for the manipulation of photoperiod to regulate the muscle quality of grass carp by evaluating the effects of photoperiod on the nutritional value and sensory quality of grass carp muscle.

## 2. Materials and Methods

### 2.1. Animal Ethics Statement

The present study was performed in accordance with the ethical guidelines laid down by the Ethics Committee of Huazhong Agricultural University (Approval date: 17 March 2024; Approval code: HZAUFI-2024-0015).

### 2.2. Fish and Rearing Conditions

The grass carp were obtained from Chonghu Fish Farm in Gong’an County, Hubei Province, China. Before the experiment, the grass carp were temporarily raised in rearing tanks (diameter of 80 cm and height of 60 cm) for 2 weeks. Subsequently, healthy grass carp with no external injuries and consistent specifications were selected for the experiment in rearing tanks of the above specifications. A commercial, expanded feed (containing 30% protein, 15% crude ash, 4% crude fat, and 12% crude fiber) (Dabeinong Technology Co., Ltd., Changde, China) was used, and the fish were fed twice daily (at 8:30 and 16:30) at 2% of the total fish body weight in each tank. Half an hour after feeding, the leftover bait was collected, and the feces were cleaned up. The body weight of the fish was assessed biweekly to adjust the amount of daily feed. During the entire aquaculture period, a continuous micro-flow aquaculture method was adopted, with dissolved oxygen in the water always being sufficient (6.0 ± 0.3 mg/L), the pH value stable (7.0 ± 0.2), the water temperature at 28 ± 0.5 °C, the nitrite nitrogen being always less than 0.01 mg/L, and the total ammonia nitrogen being always less than 0.1 mg/L.

### 2.3. Randomized Controlled Trial Design

Fifteen domesticated grass carp (whose initial mean weight was 109.65 ± 3.62 g and initial mean body length was 18.56 ± 0.29 cm (mean ± SEM)) were randomly placed in each tank for a trial period of 75 days. Five different photoperiod treatments were arranged: 0 h of light (L): 24 h of darkness (D) (continuous darkness); 8L:16D (8:00–16:00 light) (short-day photoperiod); 12L:12D (8:00–20:00 light); 16L:8D (8:00–24:00 light) (long-day photoperiod); and 24L:0D (continuous light). Three tanks were provided for each treatment. Each tank was fitted with a white light-emitting diode (LED) (12W, Tianlong Lighting Electric Appliance, Corp., Changzhou, China), which was regulated by digital clock controllers, and the light intensity was 150 lux at the bottom of each tank. A blackout cloth was used between treatments for cover and to form closed compartments to avoid cross-experimental errors caused by light sources.

### 2.4. Growth Parameters Analysis

All of the individuals were fasted for 24 h before sampling. In the sampling process, fifteen individuals were taken from each tank and anesthetized using a 200 mg/L tricaine methane sulfonate (MS-222, Argent Chemical Laboratories Inc., Redmond, WA, USA) solution to analyze the growth parameters of each fish. The final weight (FW), final length (FL), condition factor (CF), hepatosomatic index (HSI), and viscerosomatic index (VSI) were considered in the evaluation of the fish growth performance. The calculation formula used was described in detail by [12].

### 2.5. Muscle Texture Properties and Color Analysis

In accordance with the methodology proposed by [11], we randomly selected 9 fish from each tank, and 3 parallel cubic muscle samples of 1 cm × 1 cm × 1 cm were taken from each fish’s back. Subsequently, the muscle texture properties were measured by TA.XTplusC texture analyzer (Stable Micro Systems, Surrey, UK).

The determination of muscle color was conducted according to the method described by [13] using a colorimeter (model CR-400, Konica Minolta, Osaka, Japan) to measure the color difference values (L*, a*, b*) of the white and red muscles of 9 fish from each tank, respectively. Finally, the whiteness value (W*) was calculated according to the formula described by [14].

### 2.6. Muscle Conventional Nutrient Content Analysis

The dorsal muscle of each fish was taken, and the content of conventional nutrients was determined according to the national standard testing methods proposed by [15]. The moisture, ash, crude protein, and crude lipid contents of the muscle samples from 9 fish of each tank were determined.

### 2.7. Muscle Hydrolyzed Amino Acid and Free Fatty Acid Analysis

In accordance with the methodology proposed by [15], hydrolyzed amino acid and free fatty acid analysis was conducted on the muscle samples of 9 fish from each tank. The results of the amino acids are expressed as the mg per 100 g wet weight of an edible portion. The amino acid score was calculated as follows: amino acid score = (mg of amino acid in 1 g test protein)/(mg of amino acid in requirement pattern) [16]. The results of the fatty acid composition are expressed as a percentage of the total fatty acid content.

### 2.8. Muscle Hydroxyproline and Collagen Content Analysis

The hydroxyproline content of the muscle samples of 9 fish from each tank was measured according to the instructions of the Hydroxyproline Content Assay Kit (Beijing Boxbio Science & Technology Co., Ltd., Beijing, China). After converting the hydroxyproline content into a percentage (%), we calculated the collagen protein content as follows: Collagen Protein Content (%) = Hydroxyproline Content (%) × 11.1 [17].

### 2.9. Muscle Histology Observation

Fresh sections of grass carp muscle from the different treatments were fixed in 4% polyformaldehyde for 48 h at 4 °C. Following fixation, the samples were paraffin embedded, sliced, and stained with hematoxylin-eosin (H&E). Subsequently, histological observation was performed using a microscope equipped with an image analysis system (Nikon, Tokyo, Japan), and histological analyses were conducted in triplicate for each group. The diameter (d = 2 × r) of each fiber was calculated from the fiber area (A) (A = π × r^2^).

### 2.10. Gene Expression Analysis

The total RNA of the muscle samples of 9 fish from each tank was extracted using RNA isoPlus (TaKaRa, Shuzo, Kyoto, Japan), and first-strand cDNA was synthesized (Yeasen, Shanghai, China). The expression levels of the genes were conducted following the process detailed in [12], and the reagent used was Hieff^®^ qPCR SYBR Green Master Mix (Low Rox Plus) (Yeasen, Shanghai, China). The housekeeping genes *ef1α* and *β-actin* were selected, the primer sequences (Table 1) were acquired from previous studies [11], and the qPCR data were analyzed by the 2^−ΔΔCt^ method [18].

### 2.11. Statistical Analysis

The data analysis was conducted using SPSS 28.0 software (IBM, Armonk, NY, USA). Normality was evaluated using the Shapiro–Wilk test, and the homogeneity of variances was verified using Levene’s test. All the data passed the normality tests and homogeneity analysis of variance. One-way analysis of variance (ANOVA) was followed by Duncan’s multiple range test, and this was performed to analyze all the data. The mean ± standard error (SEM) was used to present the data, and *p* < 0.05 was considered statistically significant.

## 3. Results and Discussion

### 3.1. Growth Parameters of Grass Carp

The photoperiod is a key environmental factor affecting fish growth. The growth parameters of grass carp under different photoperiod conditions were compared, and the results are shown in Table 2. Regarding the FW, Treatments 12L:12D and 16L:8D exhibited higher values than Treatments 0L:24D and 8L:16D (*p* < 0.05), while Treatment 24L:0D showed intermediate values. Regarding the FL, Treatments 12L:12D, 16L:8D, and 24L:0D exhibited higher values than Treatment 0L:24D (*p* < 0.05), while Treatment 8L:16D showed intermediate values. In relation to the CF, Treatment 0L:24D exhibited higher values than Treatment 24L:0D (*p* < 0.05), while the other three treatments showed intermediate values. Consequently, this study demonstrated that appropriate photoperiod (12L:12D and 16L:8D) improved the growth parameters of grass carp. The previous study indicated that the best growth performance of zebrafish (*Danio rerio*) [19] was obtained under 16L:8D. The 16L:8D photoperiod significantly improved the growth performance of blunt snout bream (*Megalobrama amblycephala*) [20] by up-regulating the transcriptional levels of growth hormone (GH) and insulin-like growth factor-I (IGF-I) in the liver. Long photoperiods (16L:8D, 20L:4D, and 24L:0D) improved the growth performance of gibel carp (*Carassius auratus*) [21]. The implementation of a long-day photoperiod resulted in enhanced growth, which was evidenced by an increase in feed intake, an improved feed conversion ratio, a higher specific growth rate, and increased survival rates [22]. The photoperiods of 8L:16D, 12L:12D, and 16L:8D have been observed to enhance the activity of digestive enzymes, mitigate oxidative stress, and foster growth in European Sea Bass (*Dicentrachus labrax* L.) [23]. Contrary to our findings, juvenile largemouth bass (*Micropterus salmoides*) [24] have demonstrated optimal growth performance under 8L:16D. Atlantic salmon [25] and giant gourami (*Trichogaster fasciata*) [26] have also grown faster and have had the highest final weight under 0L:24D. The 0L:24D photoperiod has had a negative impacts on the growth performance of Nile tilapia (*Oreochromis niloticus*) [27]. However, several studies have reported that altering the photoperiod did not significantly affect the growth of certain fish species, including juvenile Nile tilapia [28], Rohu (*Labeo rohita*) [29], and Barramundi (*Lates calcarifer*) [30]. This indicates that the differences in fish growth performance responses to the photoperiod may originate from variations in photoperiod exposure duration, species, or stages of development. It is noteworthy that the GH/TSH/deiodinase/thyroid hormone system in the pituitary and brain, as a photoperiodic signaling system during the course of fish growth, exerts an important regulatory function on its growth [22].

### 3.2. Textural Parameters and Color of Muscle

#### 3.2.1. Textural Parameters of Muscle

**Figure 1:**
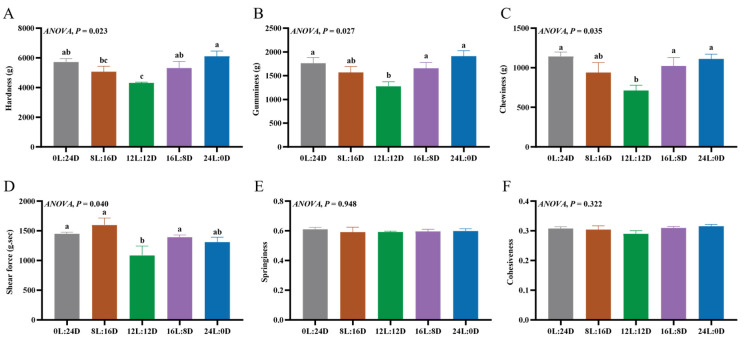
The effects of different photoperiods on the textural parameters of the muscle of grass carp (*Ctenopharyngodon idella*). (**A**) Hardness; (**B**) gumminess; (**C**) chewiness; (**D**) shear force; (**E**) springiness; and (**F**) cohesiveness. The values of the same or no superscript letters had no significant differences (*p* > 0.05).

No significant differences were shown in the springiness and cohesiveness among all groups (Figure 1E,F). Compared to the 12L:12D group, the hardness, gumminess, and chewiness in the 0L:24D, 16L:8D, and 24L:0D groups were significantly higher (*p* < 0.05) (Figure 1A–C). Hardness in the 24L:0D group was significantly higher than in the 8L:16D group (*p* < 0.05) (Figure 1A). Regarding shear force, the values of the 0L:24D, 8L:16D, and 16L:8D groups was significantly higher than that of 12L:12D group (*p* < 0.05) (Figure 1D). The texture of fish is closely related to taste, tissue morphology, and other sensory qualities [31]. Hardness is the resistance of a sample to compression at a given deformation rate [32]. Gumminess reflects the energy required to break down a colloidal food to a chewable state, and it is numerically represented as the product of hardness and cohesiveness. Chewiness reflects the energy required for a food to transition from a chewable state to a swallowable state [31]. The texture of muscle is closely related to muscle fiber characteristics, collagen content, etc. [33]. It has been observed that Shi Drum (*Umbrina cirrosa* L), when subjected to 16L:8D and 24L:0D treatments, exhibits higher hardness, gumminess, and chewiness when compared to those undergoing the 12L:12D treatment, and the white muscle fiber density of the 16L:8D group was the highest [34]. There was a positive correlation between the textural parameters and muscle fiber density [34,35]. The photoperiod had little effect on the texture of turbot, but there was a trend of increased shear force and hardness in the 24L:0D group [36].

#### 3.2.2. Color of Muscle

The color of meat is a direct indicator of quality, reflecting its freshness, and it also influences a consumer’s purchasing decisions [37]. The color of meat is influenced by the intake of carotenoids, and animals cannot synthesize this substance on their own [38]. In this study, the L* and W* values of the white muscle in the 12L:12D group were significantly higher than those in the 0L:24D and 24L:0D groups (*p* < 0.05), and the 8L:16D and 16L:8D groups exhibited intermediate values when compared with the other treatments (Table 3). There was no significant difference in the L* and W* values of the red muscle under different light cycles. The larger the L* and W* value, the lighter and whiter the fish meat, and the better the sensory visual effect. The a* value of the red muscle in the 12L:12D group was significantly lower than that in the 0L:24D, 16L:8D, and 24L:0D groups, and the 8L:16D group showed intermediate values. The larger the a* value, the closer the fish meat was to red. Extending the duration of illumination reduced the b* value of the grass carp’s white muscle. However, the b* value of the red muscle in the 12L:12D group was significantly lower than that of the other four treatments (*p* < 0.05). The larger the b* value, the closer the fish meat was to yellow, and studies have shown that fish meat leaning toward yellow is of inferior quality [39]. At present, there are few studies on the effect of photoperiod on meat color. Nevertheless, no significant effect of photoperiod was found on the muscle color of farmed cod [5].

### 3.3. Common Nutritional Component Contents of Muscle

**Figure 2:**
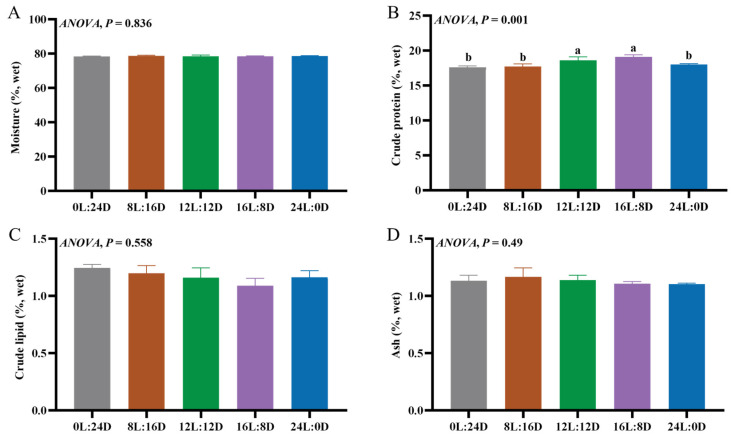
The effects of different photoperiods on the common nutritional component contents in the muscle of grass carp (*Ctenopharyngodon idella*). (**A**) The moisture contents in muscle; (**B**) the crude protein contents in muscle; (**C**) the crude lipid contents in muscle; and (**D**) the ash contents in muscle. The values of the same or no superscript letters had no significant differences (*p* > 0.05).

The primary nutritional components of fish meat are moisture, protein, and lipid [40]. There was no significant difference in the contents of crude ash, moisture, and crude lipid among the groups (Figure 2A,C,D). The content of the crude protein in the 12L:12D and 16L:8D groups was significantly higher than that in the 0L:24D, 8L:16D and 24L:0D groups (*p* < 0.05) (Figure 2B). The optimal crude protein content in the muscle of blunt snout bream was observed in the 16L:8D group, followed by the 12L:12D group, and then the 8L:16D group last, which aligns with our findings [20]. Studies have shown that the photoperiod significantly affects the crude protein content in the muscles of gibel carp when compared with the 16L:8D group, and the crude protein content in the 20L:4D group has been found to significantly increase [21]. However, when compared with a natural photoperiod, continuous illumination has no effect on the dry matter, protein, and lipid concentrations in the muscles of Atlantic cod [6]. The differences in these results may be attributed to the varying responses of the metabolism of different fish species to the photoperiod, including digestive processes. Amino acids are important components of protein. Upon digestion, food proteins are deconstructed into peptides and amino acids, which are absorbed and serve as the primary source of amino acids for fish muscle tissue [41]. The photoperiod plays a significant role in influencing the digestive enzymes’ activity in European sea bass, and trypsin and pepsin activity have been found to be higher following 24L:0D, 16L:8D, and 12L:12D treatments rather than 0L:24D and 8L:16D treatments [42]. We hypothesize that our result could potentially be attributed to the effect of the photoperiod on the protein digestion and absorption capacity of intestine, subsequently affecting the protein content within the muscles.

### 3.4. Amino Acid and Fatty Acid Composition of Muscle

#### 3.4.1. Amino Acid Composition of Muscle

**Figure 3:**
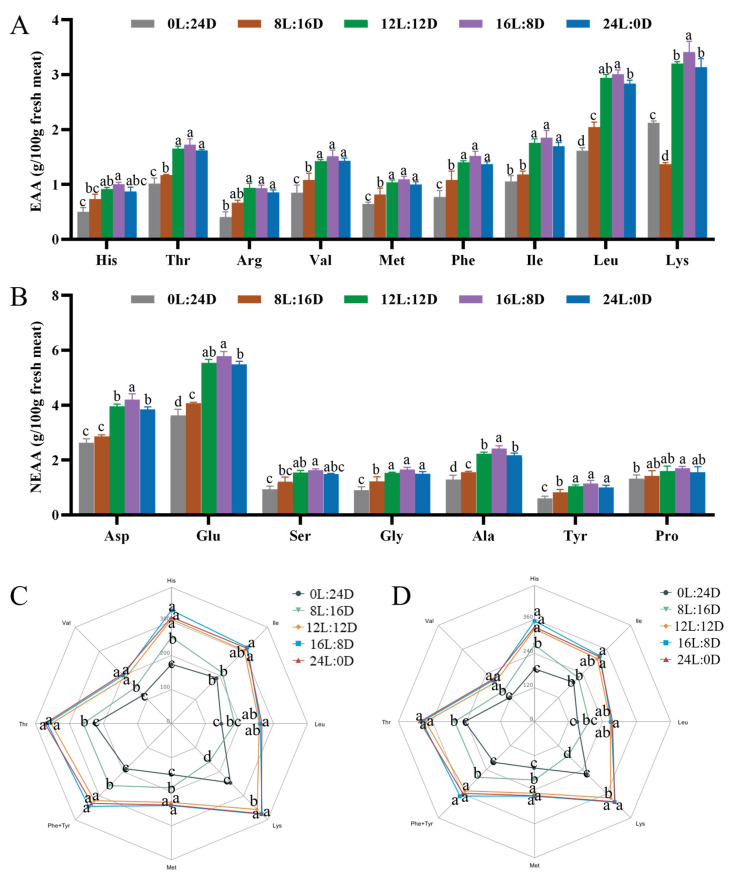
The effects of different photoperiods on the muscle amino acid compositions and amino acid chemical score of grass carp (*Ctenopharyngodon idella*). (**A**) The content of essential amino acids (EAA) in muscle; (**B**) the content of nonessential amino acids (NEAA) in muscle; (**C**) the recommended EAA chemical scoring patterns for children 3–10 years old (% of scoring pattern); and (**D**) the recommended EAA chemical scoring patterns for adults (>18 years old) (% of scoring pattern). The values of the same or no superscript letters had no significant differences (*p* > 0.05).

The quality of the proteins in muscle depends on the composition of amino acids, which significantly influence the growth, development, and nutritional quality of muscle [43,44]. In this experiment, a total of 16 amino acids were discovered in the muscles of all the groups (Figure 3). Compared with the 0L:24D group, the contents of EAAs in both the 12L:12D and 16L:8D groups were significantly increased (*p* < 0.05), and, except for histidine (His), the content of the other EAAs in the 24L:0D group were also significantly increased (*p* < 0.05) (Figure 3A). The contents of aspartic acid (Asp), glutamic acid (Glu), glycine (Gly), alanine (Ala), and tyrosine (Tyr) in the fish muscle of the 12L:12D, 16L:8D and 24L:0D groups were significantly higher than those in the 0L:24D and 8L:16D groups (*p* < 0.05) (Figure 3B). In the 16L:8D treatment, the contents of lysine (Lys), Asp, and Ala were the highest (*p* < 0.05). Similar results have been reported in Murray cods (*Maccullochella peelii*), and the concentrations of Tyr, Glu, valine (Val), and Lys in the muscle attained their peak values with the 15L:9D treatment [45]. Meanwhile, the chemical score of EAAs serves as a measure of nutritional quality, indicating the relationship between the content of EAAs in meat and the human daily requirements [16]. In contrast to the 0L:24D and 8L:16D groups, the chemical scores of threonine (Thr), His, Val, methionine (Met), Lys, and phenylalanine (Phe)+Tyr in the grass carp muscle for children (Figure 3C) and adults (Figure 3D) were significantly increased in the 12L:12D, 16L:8D, and 24L:0D groups (*p* < 0.05). The chemical scores of isoleucine (Ile) and leucine (Leu) in the 24L:0D group were significantly higher than those in the 0L:24D and 8L:16D groups (*p* < 0.05). The results indicate that a suitable photoperiod could enhance the nutritional value of grass carp meat, thus meeting the demand for EAAs for humans.

#### 3.4.2. Fatty Acid Composition of Muscle

**Figure 4:**
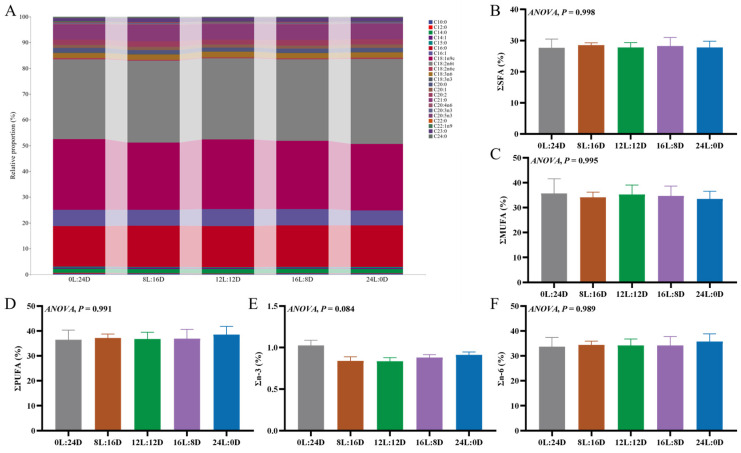
The effects of different photoperiods on the muscle fatty acid composition of grass carp (*Ctenopharyngodon idella*). (**A**) Interactive ribbon bar chart; (**B**) Σsaturated fatty acid (ΣSFA); (**C**) Σmonounsaturated fatty acids (ΣMUFA); (**D**) Σpolyunsaturated fatty acids (ΣPUFA); (**E**) Σn-3 fatty acid (Σn-3); and (**F**) Σn-6 fatty acids (Σn-6).

In this study, a total of 23 fatty acids were discovered. The primary fatty acid composition of the grass carp muscle in five photoperiod treatments were C18:2n6t, C18:1n9c, and C16:0 (Figure 4A). Compared to the 0L:24D group, the content of C12:0 was lower in the 12L:12D and 24L:0D groups (Table 4). The content of C14:1 in the 8L:16D group was higher than that in the 12L:12D group. No significance was found in the ΣSFA, ΣMUFA, ΣPUFA, Σn-3, and Σn-6 among all the photoperiod groups (Figure 4B–F). The findings of this study align with those of studies on grass carp [46], *Silurus meridionalis* [15], and European seabass [8] in terms of the fatty acid composition in muscle tissue. In these studies, C16:0 emerged as the predominant SFA, C18:1n9 as the primary MUFA, and C18:2n6 as the leading PUFA. For European seabass, the photoperiod did not significantly influence the MUFA content in its muscles. However, the concentrations of C14:0 and SFA in the muscle of European seabass were notably higher under the 12L:12D and 16L:8D photoperiod conditions compared to the 0L:24D and 24L:0D conditions. Conversely, the PUFA content in the 24L:0D group was markedly greater than that in the 0L:24D and 8L:16D groups. A prolonged photoperiod might induce stress in European seabass, prompting increased energy expenditure [8]. In contrast, our study suggests that a grass carp’s fatty acid composition is minimally affected by the photoperiod, implying that a long-term photoperiod does not induce significant stress in grass carp.

### 3.5. The Main Muscle Components Involved in the Development of Flesh Texture

Within the various components of skeletal muscle, three primary factors are recognized as determinants of overall muscle properties, including the following textural characteristics: muscle fibers, intramuscular connective tissue, and intramuscular fat [47]. In the present study, no significant differences were produced in the muscle fat content of the grass carp in all photoperiods (Figure 2C). In the subsequent section, we will explore the influence of the photoperiod on the properties of grass carp muscle fibers and collagen synthesis.

#### 3.5.1. Muscle Fiber Properties

**Figure 5:**
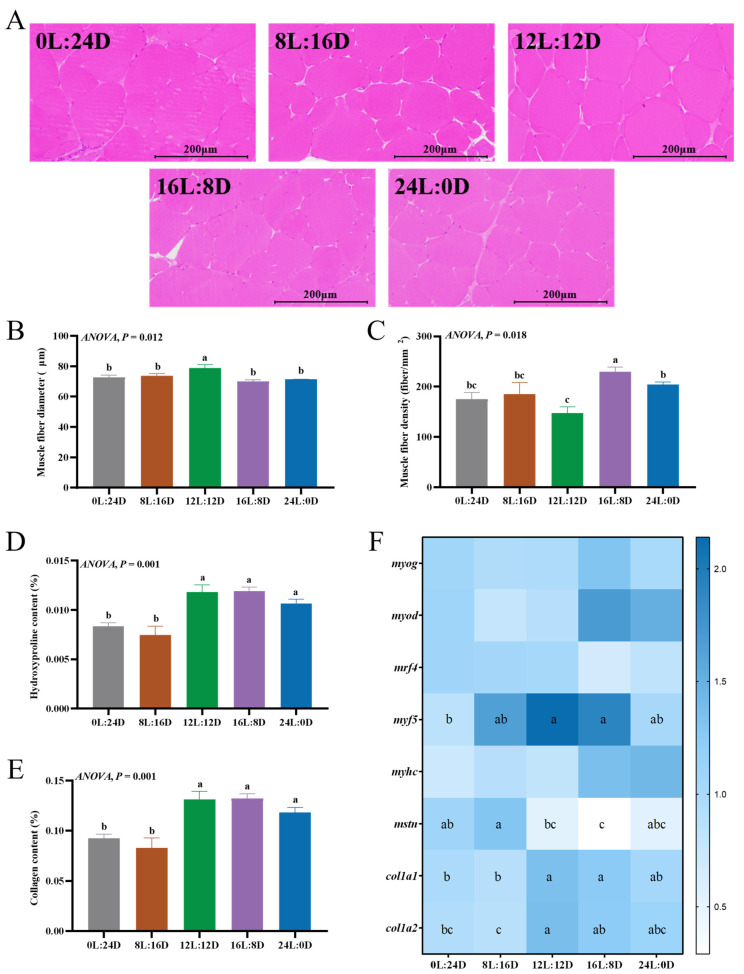
The effects of different photoperiods on the main muscle components involved in the development of flesh texture in grass carp (*Ctenopharyngodon idella*). (**A**) Muscle fiber microstructure visualized using hematoxylin-eosin staining; (**B**) muscle fiber diameter; (**C**) muscle fiber density; (**D**) the hydroxyproline contents in muscle; (**E**) the collagen contents in muscle; and (**F**) the mRNA levels of muscle fiber-related genes and collagen synthesis-related genes. The values of the same or no superscript letters had no significant differences (*p* > 0.05).

The fundamental structural component of skeletal muscle is muscle fiber [48]. In this study, the photoperiod had significant effects on the grass carp muscle fiber properties (Figure 5A–C). The 12L:12D group had the highest muscle fiber diameter (*p* < 0.05) (Figure 5B). The 16L:8D group had significantly higher muscle fiber density than the other groups, and the 12L:12D group had significantly lower myofiber density than the 24L:0D group (*p* < 0.05) (Figure 5C). Distinct from terrestrial animals, the majority of fish species exhibit continuous muscle growth throughout adulthood, which is attributed to a combination of hyperplasia (formation of new muscle fibers) and hypertrophy (increase in fiber size) [49]. Therefore, the increase in muscle fiber diameter and decrease in the density in the 12L:12D group may be due to muscle fiber hypertrophy, while the muscle fiber characteristics of the 16L:8D group may be caused by muscle fiber hyperplasia. The smaller the fiber diameter, the higher the density, the firmer the muscle, and the higher the hardness [2,50,51]. As the cross-sectional area of fish muscle fibers increases, the hardness of the fish meat decreases [52]. Therefore, we speculated that the lowest hardness of the grass carp under the 12L:12D group may be due to the increase in muscle fiber diameter in this group.

The process of myogenesis in vertebrates involves the action of various signaling molecules, among which myogenic regulatory factors (MRFs), such as *myod*, *myf5*, *myog*, and *mrf4*, receive significant attention [53]. Myostatin (*mstn*) inhibits myogenesis by suppressing the expression of MRFs [54]. In this study, the *mstn* and *myf5* mRNA levels in the muscle were significantly affected by the photoperiod (*p* < 0.05) (Figure 5F). The 12L:12D and 16L:8D groups had significantly higher *myf5* mRNA levels than the 0L:24D group. The 8L:16D group had a significantly higher *mstn* mRNA level than the 12L:12D and 16L:8D groups. The 0L:24D group had a significantly higher *mstn* mRNA level than the 16L:8D group. Previous research has demonstrated that, in comparison to a natural photoperiod, continuous light of 120 days enhances the growth of Atlantic cod and significantly elevates the *myog* mRNA levels in muscle. However, the impact on *myf5* mRNA levels is not significant [23]. In the case of Atlantic salmon, a photoperiod treatment of 2 months significantly influences the levels of MRFs in muscle, with *myog* mRNA levels increasing significantly as the photoperiod extends. Conversely, the *myf5* mRNA levels in the muscle were significantly reduced in the 16L:8D and 24L:0D groups when compared to a natural photoperiod [25]. Nevertheless, a photoperiod treatment of 5 months did not significantly affect the *myhc*, *myf5*, and *myog* mRNA levels in the muscle of Atlantic salmon, while the *myod* and *mstn* mRNA levels were significantly higher in the natural photoperiod group than in the continuous light group [55]. The variation in these results may be attributed to differences of species, as well as to the differential response of the same species at various life stages. *Myog* and *mrf4* are key genes in myofiber differentiation, while *myod* and *myf5* not only regulate myofiber differentiation, but also promote muscle cell proliferation in mice [56]. The findings of this study suggest that the photoperiod may primarily influence muscle fiber growth by augmenting *myf5* expression and suppressing *mstn* expression.

#### 3.5.2. Collagen Synthesis

Collagen is a key component of intramuscular connective tissue and is essential for maintaining tissue structure and strength [57]. The content of collagen is determined by the hydroxyproline content [58]. In this study, the contents of hydroxyproline (Figure 5D) and collagen (Figure 5E) in the 0L:24D and 8L:16D groups were significantly lower than that in the 12L:12D, 16L:8D, and 24L:0D groups (*p* < 0.05). A previous study has shown that the photoperiod conditions of 8L:16D, 12L:12D, and 16L:8D have enhanced the collagen content in the muscle of European seabass. However, no such effect was observed in the group subjected to a 24L:0D photoperiod [8]. Type I collagen is the main component of fish muscle connective tissue collagen [59], and it is primarily orchestrated by *col1α1* and *col1α2* [31]. In the current study, the mRNA level of *col1α1* in the 12L:12D and 16L:8D groups was significantly higher than that in the 0L:24D and 8L:16D groups (*p* < 0.05). The mRNA level of the *col1α2* in the 12L:12D group was significantly higher than that in the 0L:24D and 8L:16D groups (*p* < 0.05), and the mRNA level of the *col1α2* in the 16L:8D group was significantly higher than that in the 8L:16D group (*p* < 0.05) (Figure 5F). Consequently, the observed increase in collagen content within the 12L:12D and 16L:8D groups could be attributed to the up-regulation of *col1α1* and *col1α2* mRNA levels, leading to an augmented synthesis of the peptide chains α1 and α2. It is generally believed that the increase in fish meat hardness is related to a rise in collagen content, but the muscle texture results of the 12L:12D group in this study did not align with this conclusion. Previous research has shown that adding cinnamaldehyde to a diet decreases the hardness of grass carp muscle while simultaneously increasing their collagen content. The decrease in muscle hardness due to cinnamaldehyde supplementation might not be related to the collagen content in grass carp muscles [9].

## 4. Conclusions

In summary, the current study revealed that a long-day photoperiod (16L:8D) treatment could effectively promote the growth, improve the nutritional value of muscle, and enhance muscle hardness, thereby optimizing the muscle quality of grass carp. The improvement of muscle nutritional value may be attributed to the positive effect of the photoperiod on the amino acid composition in muscle. Moreover, the enhancement of muscle hardness might be associated with myofiber development and collagen biosynthesis. These findings not only provide a theoretical support for how the photoperiod improves the muscle quality of grass carp, but they are also valuable data for the research field of food science.

## Figures and Tables

**Table 1:** The primer list of this study.

Gene Name	Primer Sequence (5′-3′)	Accession Numbers
*mrf4*	F: TCGCTCCTGTATTGATGTTGATG	KT899334
	R: GCTCCTGTCTCGCATTCGT	
*myf5*	F: GGAGAGCCGCCACTATGA	GU290227
	R: GCAGTCAACCATGCTTTCAG	
*myog*	F: CGGCGATAACTTCTTCCA	JQ793897
	R: TTCTTCAACCTCCTCTTCTC	
*myod*	CGCTACTTAGGAGTCAAGAGGA	GU218462
	AGTTCTCACCATGCCATCAGA	
*mstn*	GCAGGAGTCACGTCTTGGCA	KM874826
	GAGTCCCTCCGGATTCGCTT	
*col1α1*	GCATGGGGCAAGACAGTCA	HM363526
	ACGCACACAAACAATCTCAAGT	
*col1α2*	ACATTGGTGGCGCGCAGATCA	HM587241
	TCTCCGATAGAGCCCAGCTT	
*ef1α*	TGACTGTGCCGTGCTGAT	GQ266394
	GCTGACTTCCTTGGTGATTTC	
*β-actin*	CCTTCTTGGGTATGGAATCTTG	DQ211096
	AGAGTATTTACGCTCAGGTGGG	

Abbreviations: *mrf4*, myogenic regulatory factor 4; *myf5*, myogenic regulatory factor 5; *myog*, myoglobin; *myod*, myogenic determinant; *mstn*, myostatin; *col1α1*, type I collagen α1; *col1α2*, type I collagen α2; *ef1α*, Elongation factor 1 alpha; and *β-actin*, beta-actin.

**Table 2:** Comparison of the growth parameters of grass carp (*Ctenopharyngodon idella*) in different photoperiods.

Items	Photoperiods				
	0L:24D	8L:16D	12L:12D	16L:8D	24L:0D
FW (g)	360.32 ± 17.41 ^b^	363.64 ± 5.85 ^b^	382.48 ± 5.84 ^a^	383.98 ± 9.3 ^a^	367.1 ± 3.84 ^ab^
FL (cm)	25.34 ± 0.4 ^b^	25.64 ± 0.13 ^ab^	26.16 ± 0.53 ^a^	26.19 ± 0.16 ^a^	25.99 ± 0.29 ^a^
CF (g/cm^3^)	2.00 ± 0.03 ^a^	1.95 ± 0.03 ^ab^	1.93 ± 0.07 ^ab^	1.92 ± 0.02 ^ab^	1.89 ± 0.08 ^b^
HSI (%)	2.23 ± 0.18	2.02 ± 0.17	2.19 ± 0.12	2.26 ± 0.21	2.02 ± 0.06
VSI (%)	9.87 ± 0.71	9.41 ± 0.58	9.63 ± 0.60	10.04 ± 0.58	9.48 ± 0.37

FW, final weight; FL, final length; CF, condition factor; HSI, hepatosomatic index; and VSI, viscerosomatic index. The values of the same or no superscript letters in the same row had no significant differences (*p* > 0.05).

**Table 3:** The effects of different photoperiod treatments on the flesh color of grass carp (*Ctenopharyngodon idella*).

Chroma Index	Photoperiods				
	0L:24D	8L:16D	12L:12D	16L:8D	24L:0D
White muscle					
L*	47.09 ± 0.14 ^b^	47.45 ± 1.03 ^ab^	50.5 ± 2.73 ^a^	48.6 ± 2.52 ^ab^	47.05 ± 0.5 ^b^
a*	−0.91 ± 0.19	−1.05 ± 0.31	−0.92 ± 0.01	−1.12 ± 0.04	−1.19 ± 0.07
b*	−1.55 ± 0.27 ^a^	−1.57 ± 0.25 ^a^	−1.81 ± 0.3 ^ab^	−2.3 ± 0.43 ^b^	−2.45 ± 0.58 ^b^
W*	47.06 ± 0.14 ^b^	47.42 ± 1.03 ^ab^	50.47 ± 2.74 ^a^	48.54 ± 2.5 ^ab^	46.97 ± 0.48 ^b^
Red muscle					
L*	40.71 ± 0.89	41.03 ± 0.81	41.34 ± 1.41	40.66 ± 0.56	41.34 ± 0.77
a*	13.88 ± 0.62 ^a^	12.58 ± 0.58 ^ab^	11.83 ± 0.56 ^b^	13.32 ± 1.18 ^a^	13.82 ± 0.51 ^a^
b*	3.36 ± 0.15 ^a^	3.38 ± 0.23 ^a^	2.81 ± 0.18 ^b^	3.55 ± 0.31 ^a^	3.45 ± 0.06 ^a^
W*	39.01 ± 0.86	39.6 ± 0.68	40.11 ± 1.48	39.07 ± 0.82	39.67 ± 0.81

L*, lightness; a*, red/greenness; b*, yellowness; and W*, whiteness. The values of the same or no superscript letters in the same row had no significant differences (*p* > 0.05).

**Table 4:** The fatty acid (FA) composition (%) of grass carp (*Ctenopharyngodon idella*) in different photoperiods.

Items	Photoperiods				
	0L:24D	8L:16D	12L:12D	16L:8D	24L:0D
C10:0	0.39 ± 0.21	0.30 ± 0.08	0.26 ± 0.02	0.37 ± 0.11	0.31 ± 0.08
C12:0	0.19 ± 0.04 ^a^	0.15 ± 0.02 ^ab^	0.10 ± 0.03 ^b^	0.14 ± 0.02 ^ab^	0.10 ± 0.03 ^b^
C14:0	1.63 ± 0.42	1.65 ± 0.16	1.74 ± 0.17	1.67 ± 0.42	1.62 ± 0.25
C14:1	0.48 ± 0.03 ^ab^	0.50 ± 0.00 ^a^	0.40 ± 0.01 ^b^	0.46 ± 0.04 ^ab^	0.46 ± 0.03 ^ab^
C15:0	0.31 ± 0.06	0.30 ± 0.01	0.32 ± 0.03	0.30 ± 0.06	0.35 ± 0.05
C16:0	15.73 ± 3.22	15.99 ± 1.24	15.96 ± 1.75	16.12 ± 2.92	16.19 ± 2.29
C16:1	6.38 ± 1.96	6.25 ± 0.77	6.64 ± 1.16	6.30 ± 1.50	5.73 ± 1.10
C18:1n9c	27.38 ± 8.02	26.05 ± 2.75	26.95 ± 5.25	26.54 ± 5.19	25.84 ± 4.26
C18:2n6t	31.01 ± 6.21	31.72 ± 2.42	31.50 ± 4.01	31.54 ± 5.70	33.06 ± 4.95
C18:2n6c	0.45 ± 0.05	0.44 ± 0.05	0.46 ± 0.06	0.39 ± 0.09	0.43 ± 0.09
C18:3n6	1.94 ± 0.30	2.01 ± 0.17	2.04 ± 0.32	1.99 ± 0.38	2.06 ± 0.31
C18:3n3	0.38 ± 0.06	0.32 ± 0.03	0.32 ± 0.06	0.32 ± 0.06	0.33 ± 0.04
C20:0	1.58 ± 0.32	1.46 ± 0.16	1.42 ± 0.25	1.47 ± 0.26	1.47 ± 0.20
C20:1	1.22 ± 0.21	1.20 ± 0.12	1.15 ± 0.16	1.25 ± 0.18	1.32 ± 0.20
C20:2	2.05 ± 0.28	2.15 ± 0.12	1.94 ± 0.27	2.08 ± 0.31	2.06 ± 0.30
C21:0	5.90 ± 0.69	6.47 ± 0.37	6.00 ± 0.48	6.08 ± 0.63	5.93 ± 0.64
C20:4n6	0.25 ± 0.03	0.20 ± 0.03	0.20 ± 0.01	0.22 ± 0.02	0.22 ± 0.01
C20:3n3	0.49 ± 0.04	0.42 ± 0.03	0.41 ± 0.03	0.45 ± 0.01	0.44 ± 0.01
C20:5n3	0.15 ± 0.03	0.10 ± 0.04	0.10 ± 0.04	0.11 ± 0.01	0.14 ± 0.03
C22:0	0.13 ± 0.04	0.10 ± 0.04	0.09 ± 0.02	0.11 ± 0.02	0.09 ± 0.02
C22:1n9	0.19 ± 0.06	0.11 ± 0.06	0.13 ± 0.05	0.09 ± 0.05	0.13 ± 0.02
C23:0	1.32 ± 0.16	1.58 ± 0.31	1.43 ± 0.32	1.54 ± 0.40	1.28 ± 0.11
C24:0	0.47 ± 0.13	0.52 ± 0.09	0.45 ± 0.10	0.43 ± 0.07	0.45 ± 0.09

The values of the same or no superscript letters in the same row had no significant differences (*p* > 0.05).

## Data Availability

The original contributions presented in this study are included in the article, and further inquiries can be directed to the corresponding author.
